# A Concepção do Escore de Selvester: Interface com o Desenvolvimento da Eletrovetorcardiografia

**DOI:** 10.36660/abc.20230584

**Published:** 2023-10-03

**Authors:** Carlos Alberto Pastore

**Affiliations:** 1 Hospital das Clínicas Faculdade de Medicina Universidade de São Paulo São Paulo SP Brasil Unidade Clínica de Eletrocardiografia, Instituto do Coração (InCor), Hospital das Clínicas FMUSP, Faculdade de Medicina, Universidade de São Paulo, São Paulo, SP – Brasil

**Keywords:** Ron Selvester, Infarto do Miocárdio, Doença Arterial Coronariana, Mortalidade, Diagnóstico por Imagem/métodos, Diagnóstico por Imagem/tendências, Eletrocardiografia, Ecocardiografia, Vetocardiografia

Na Conferência Anual da *International Society for Computerized Electrocardiology* (ISCE) em 2015, em San José – Costa Rica foi organizada uma sessão para homenagear o extraordinário trabalho e a vida de especialistas em eletrocardiologia, entre eles, Ron Selvester.

Ron Selvester foi um dos melhores editores do *Journal of Eletrocardiology*, recrutou os melhores conhecedores da área para serem coeditores, e a Revista cresceu em conteúdo e fator de impacto. Foi pioneiro no campo da pesquisa, um visionário extraordinário. Ron foi o primeiro a mensurar como um infarto do miocárdio poderia ser diagnosticado em princípio com bloqueio de ramo, critério para intervir e dimensionar infarto.

Em 1975, a *International Business Machines* (IBM) construiu um caminho digital que lia ECGs, permitindo reproduzir o trabalho dos cardiologistas.

A IBM estava mirando em um alvo móvel, tentando continuamente sugerir os seus critérios. Posteriormente, a *Food and Drug Administration* (FDA) foi obrigada por lei a certificar a medição das máquinas que liam ECG. Dessa forma, em 1975, o FDA reuniu um grupo no qual estava Ron Selvester com a incumbência de resolver de uma vez por todas os critérios para leitura dos ECGs. O InCor da FMUSP, inaugurado em 1977 recebeu os aparelhos da Hewlett-Packard (HP) com algoritmos definidos por esse grupo, mas infelizmente não foi possível utilizá-los, pois não eram compatíveis com nossa análise e experiência.

Ron Selvester foi um grande defensor do Vetorcardiograma, pela distribuição dos eletrodos deste exame ser mais completa e reproduzir de forma mais eficaz o fenômeno espacial e elétrico.^1^

A *International Society of Electrocardiology* (ISE) teve seu início nos Colóquios de Vetorcardiograma em 1959. Em 1973 esses Colóquios começaram a ser apresentados no *International Congress on Electrocardiology* (ICE).

Após vários congressos, 10 anos, o Conselho Internacional integrou a ISE. Ron Selvester foi o Presidente de 1995 a 1997. Eu tive a honra de ser o primeiro representante do Brasil como Presidente do Conselho da *International Society of Eletrocardiology* de 2013 a 2015.

No Congresso da ISCE em 1982, Ron apresentou a conferência “Validação patológica dos Critérios Computacionais para localização do infarto em 12 segmentos do ventrículo esquerdo”.

No ano de 1985 já publicava no *ARCH International* o clássico trabalho: “*The Selvester QRS Scoring System for Estimating Myocardial Infarct size. The Development and Application of the System”.*^2^

Neste artigo original^3^ os autores buscam mostrar que o escore de Selvester pode ter valor preditivo na mortalidade da insuficiência cardíaca com fração de ejeção preservada.

Foram investigados 359 pacientes. O escore de Selvester simplificado foi mantido e registrado.^3^ Os ECGs foram pontuados manualmente de acordo com o sistema simplificado de pontuação de 37 critérios e 29 pontos de Bounous et al.,1988.^4^ A pontuação S-QRS foi calculada manualmente por dois cardiologistas experientes, levando em consideração um algoritmo relatado anteriormente. Em caso de não concordância entre o laudo dos dois profissionais, um terceiro cardiologista calculava o escore do S-QRS de forma cega e o finalizava. O sistema de pontuação foi baseado em critérios para 10 das 12 derivações de um ECG padrão (aVL, aVF, I, II, V1-6). Os pontos são dados para a duração da onda Q, amplitudes e duração R e relação R/S ou R/Q.^3^

O escore S-QRS fornece informações sobre o tamanho e a localização das cicatrizes miocárdicas, examinando as alterações morfológicas do QRS que ocorrem devido às alterações da despolarização ventricular resultantes da fibrose miocárdica. Uma correlação expressiva entre pontuação S-QRS e o tamanho da cicatriz foi encontrada em diversos estudos de autópsia e ressonância magnética. Em estudo prospectivo de Liu Q. et al.,^5^ onde 289 pacientes foram acompanhados por 2 anos após infarto do miocárdio com elevação do segmento ST, observou-se que a mortalidade cardiovascular aumentou 1,46 vezes em pacientes com escores S-QRS elevados, quando comparado com pacientes que não o fizeram.

O escore também se mostrou um fator de risco independente para a mortalidade (sensibilidade de 80,8%, especificidade de 77,2% e corte de 5,5). Na análise verificou-se maior mortalidade no grupo com escore de Selvester acima ou igual a 5,5, em relação ao grupo com escore abaixo desse valor.^3^

O escore S-QRS aferido pelo ECG padrão de 12 derivações foi considerado um fator de risco independente para mortalidade em pacientes com insuficiência cardíaca com fração de ejeção preservada (ICFEP). Portanto, fornece informações sobre a mortalidade do paciente mesmo na ausência de acesso à RM cardíaca, ou quando outros parâmetros de ECG são normais. Assim, recomendamos que o escore S-QRS não seja negligenciado na avaliação de pacientes de alto risco.^3^

O escore de Selvester vem sendo aplicado nas mais variadas situações clínicas, e ainda utilizado como referência na literatura.

O importante deste minieditorial é resgatar os caminhos da eletrocardiologia e como esses grupos de cientistas trabalharam em alto nível, mesmo com os poucos recursos da época. Estas Sociedades formadas por estudiosos, médicos, bioengenheiros e eletrofisiologistas trouxeram uma contribuição excepcional para cardiologia.
