# Supplementary material for: A Concepção do Escore de Selvester: Interface com o Desenvolvimento da Eletrovetorcardiografia
Source: Arq Bras Cardiol. 2023 Oct 3;120(9):e20230584. [Article in Portuguese] doi: 10.36660/abc.20230584 (PMC10547434; doi:10.36660/abc.20230584)
Supplement: Translation

# Translation: The Conception of the Selvester Score: Interface with the Development of Electro-Vectorcardiography

**Authors:** Carlos Alberto Pastore

**Affiliations:** 1 Brasil Unidade Clínica de Eletrocardiografia, Instituto do Coração (InCor), Hospital das Clínicas FMUSP, Faculdade de Medicina, Universidade de São Paulo, São Paulo, SP – Brasil

**Keywords:** Ron Selvester, Myocardial Infarction, Coronary Artery Diseases Mortality, Diagnostic, Imaging/trends, Electrocardiography, Echocardiography, Vetocariography

At the Annual Conference of the International Society for Computerized Electrocardiology (ISCE) in 2015, in San José – Costa Rica, a session was organized to honor the extraordinary work and life of specialists in electrocardiology, among them Ron Selvester.

Ron Selvester was one of the best editors of the Journal of Electrocardiology. He recruited the best connoisseurs to be co-editors, leading the Journal to grow in content and improving its impact factor.

Dr. Selvester was a pioneer in the research field and an extraordinary visionary. He was among the first to propose a measurement method for acute myocardial infarction in the presence of a left bundle-branch block (Selvester 10% rule) and a criterion for dimensioning myocardium infarction scar with and without confounders such as bundle-branch blocks.

In 1975, International Business Machines (IBM) built a digital pathway that read ECGs, allowing it to reproduce the work of cardiologists. IBM was aiming at a moving target, continually trying to suggest its criteria. Subsequently, the Food and Drug Administration (FDA) was legally required to certify the measurement of machines that read ECGs. Thus, in 1975, the FDA assembled a group in which was Ron Selvester, with the task of solving once and for all the criteria for reading ECGs. The *Instituto do Coração* (InCor FMUSP), inaugurated in 1977, received Hewlett-Packard (HP) devices with algorithms defined by this group. Still, unfortunately, it was not possible to use them, as they were incompatible with our analysis and experience.

Ron Selvester was a great advocate of the Vectorcardiogram, as the distribution of the electrodes of this exam is complete and reproduces more effectively the spatial and electrical phenomenon.^1^

The International Society of Electrocardiology (ISE) began at the Vectorcardiogram Colloquium in 1959. In 1973 these colloquiums started to be invited and exposed at the International Congress on Electrocardiology (ICE).

After several congresses (ten years), the International Council joined ISE. Ron Selvester was President from 1995 to 1997. I had the honor of being the first representative of Brazil as President of the International Society of Electrocardiology from 2013 to 2015.

At the ISCE Congress in 1982, Ron presented the conference “Pathological Validation of the Computational Criteria for Locating the Infarction in 12 Segments of the left ventricle”.

In 1985 he was already publishing in ARCH International the classic work: “The Selvester QRS Scoring System for Estimating Myocardial Infarct size. The Development and Application of the System”.^2^

In this original article,^3^ the authors seek to show that the Selvester score may have predictive value in the mortality of heart failure with preserved ejection fraction.

A total of 359 patients were investigated. The simplified Selvester score was maintained and recorded.^3^ The ECGs were manually scored according to the simplified scoring system of 37 criteria and 29 points of Bounous et al.,1988.^4^ The S-QRS score was manually calculated by two qualified cardiologists, taking into account a previously reported algorithm. In case of non-agreement between the report of the two professionals, a third cardiologist calculated the S-QRS score blindly and finalized it. The scoring system was based on criteria for 10 of the 12 leads of a standard ECG (aVL, aVF, I, II, V1-6). Points are given for Q wave duration, R amplitudes, and duration, and R/S or R/Q ratio.^3^

The S-QRS score provides information on the size and location of myocardial scars, examining the morphological changes in QRS that occur due to ventricular depolarization changes resulting from myocardial fibrosis. Several autopsies and magnetic resonance imaging studies found a significant correlation between the S-QRS score and scar size. In a prospective survey by Liu Q. et al.^5^ where 289 patients were followed for two years after myocardial infarction with ST-segment elevation, it was observed that cardiovascular mortality increased 1.46 times in patients with elevated S-QRS scores when compared to patients who did not.

The score also proved to be an independent risk factor for mortality (sensitivity of 80.8%, specificity of 77.2%, and cut-off of 5.5). The analysis found higher mortality in the group with a Selvester score above or equal to 5.5 compared to the group with a score below this value.^3^

The S-QRS score measured by the standard 12-lead ECG was considered an independent risk factor for mortality in patients with heart failure with preserved ejection fraction (HFpEF). Therefore, it provides information on patient mortality even without access to cardiac MRI or when other ECG parameters present a normal range. Thus, the S-QRS score should not be neglected in evaluating high-risk patients.^3^

The Selvester score has been used in the most varied clinical situations and is still used as a reference in the literature.

This short editorial aim to rescue the paths of electrocardiology and how these groups of scientists worked at a high level, even with the few resources of the time. These Societies formed by scholars, physicians, bioengineers, and electrophysiologists brought an exceptional contribution to cardiology.
